# Genetic analysis of High Immune Response technology and relationships with disease resilience in pigs

**DOI:** 10.1093/jas/skag163

**Published:** 2026-05-20

**Authors:** Jian Cheng, Julie Schmied, Bonnie Mallard, John C S Harding, Michael K Dyck, Frederic Fortin, Graham S Plastow, PigGen Canada, Jack C M Dekkers

**Affiliations:** Department of Animal Science, Iowa State University, Ames, Iowa 50011, United States; Department of Pathobiology, Ontario Veterinary College, University of Guelph, Guelph, Ontario, Canada N1G 2W1; Department of Pathobiology, Ontario Veterinary College, University of Guelph, Guelph, Ontario, Canada N1G 2W1; Department of Large Animal Clinical Science, University of Saskatchewan, Saskatoon, Saskatchewan, Canada S7N 5A2; Department of Agriculture, Food and Nutritional Science, University of Alberta, Edmonton, Alberta, Canada T6G 2R3; Centre de Développement du Porc du Québec Inc, Québec City, Québec, Canada G1V 4M6; Department of Agriculture, Food and Nutritional Science, University of Alberta, Edmonton, Alberta, Canada T6G 2R3; PigGen Canada Research Consortium, Guelph, Ontario, Canada N1H4G8; Department of Animal Science, Iowa State University, Ames, Iowa 50011, United States

**Keywords:** immune response, disease resilience, genetics, pigs, major histocompatibility complex, high immune response

## Abstract

Disease resilience is a viable target for genetic improvement programs in pigs to improve productivity and animal welfare and reduce the use of antibiotics. However, disease resilience is difficult to select for in nucleus breeding programs. Indicator traits that can be measured on (young) pigs without exposure to disease offer possible solutions. The High Immune Response (HIR) technology has been shown to provide such indicator traits in dairy cattle. HIR involves evaluation of antibody-mediated (AMIR) and cell-mediated (CMIR) immune responses. The objectives of this study were to implement HIR technology for young and clinically healthy pigs, to evaluate genetic parameters of, and genomic regions associated with AMIR and CMIR, and estimate genetic correlations of AMIR and CMIR with disease resilience. For the latter, data from a polymicrobial natural disease challenge that pigs were exposed to after HIR evaluation were used. In total, HIR data were collected on 2,295 Yorkshire × Landrace barrows, as well as disease resilience data on these and another 1,800 barrows from the same populations. All pigs had genotypes for 451,389 single-nucleotide polymorphisms (SNPs) across the genome. Both AMIR and CMIR were moderately heritable (0.25 ± 0.06 and 0.20 ± 0.05), with limited evidence of a genetic correlation between them (0.23 ± 0.17). Higher AMIR tended to be genetically correlated with higher growth and health under disease, but the opposite was apparent for CMIR, possibly as a result of the nature of the disease challenge and the clinical disease data that was collected, which tended to emphasize bacterial rather than viral infections. Genetic correlations of AMIR and CMIR with disease resilience traits were also not consistent between two sets of batches of pigs separated in time but this appeared to be the result of genetic differences in disease resilience, possibly because of different disease dynamics, rather than in HIR traits, as the estimate of the genetic correlation between the two sets of batches was low for growth under challenge (0.44 ± 0.38) but high for AMIR (0.86 ± 0.35) and CMIR (0.91 ± 0.48). The major histocompatibility complex was found to explain over 32% of the genetic variation for AMIR, with SNP AX-116767878 explaining the majority of this variation, and was also associated with disease resilience. Three other genomic regions were identified for AMIR, together explaining 12% of genetic variance, while only two regions were identified for CMIR, each explaining less than 2% of genetic variance. All identified regions included genes associated with the immune response. Results suggest that HIR traits, in particular AMIR, are strong candidates as indicator traits that can be measured on young healthy pigs to select for resilience across multiple pathogens.

## Teaser

The High Immune Response (HIR) technology, which has been pioneered in dairy cattle, also provides heritable measures of antibody-mediated and cell-mediated immune response. Antibody-mediated immune response was found to be an indicator trait to select disease resilience in pigs because it can be evaluated on young and clinically healthy selection candidates without exposure to disease.

## Introduction

Infectious diseases provide an ongoing challenge for the pork industry, resulting in tremendous financial losses, reduced animal welfare, and greater use of antibiotics. Integration of selection for immune response capacity in animal breeding programs has long been suggested as an effective method to reduce the impact of infectious diseases (Wilkie and Mallard 1999; Abdel-Azim et al. 2005; Mallard et al. 2011; Knap and Doeschl-Wilson 2020; Shi et al. 2025). This can be based on selecting for host resistance against specific pathogens or breeding for broad-based disease resistance to a diverse array of pathogens. In pigs and other livestock, research has mainly focused on the former and this has yielded variable results. For example, resistance to post-weaning diarrhea caused by F18+ *Escherichia coli* in pigs is linked to a single nucleotide change. Selecting for this marker therefore provides resistance to specific bacteria, which can reduce mortality and improve growth rates on affected farms (Abdel-Azim et al. 2005). However, this approach is limiting as it confers resistance to a single specific pathogen amongst many that are capable of causing porcine diarrhea. Other research groups have attempted to identify naturally occurring resistance genes to other important pathogens of swine but with limited success. For example, Torricelli et al. (2024) investigated an interleukin-18 gene polymorphism as a potential marker for resistance to the parasite *Toxoplasma gondii*, a pathogen of swine that poses a significant food-safety risk to humans. The study, however, revealed significant associations only in seropositive animals. Likewise, as host defenses evolve, so do pathogen virulence mechanisms, as observed recently in farmed Atlantic Salmon that were selected for resistance to infectious pancreatic necrosis virus (IPNV) based on a previously identified natural mutation in a major gene for resistance (Robledo et al. 2016). Since the resistance conferred by the identified mutation was mediated by a more effective immune response to the IPNV virus, not the absence of a receptor critical for infection as above, selective pressure imposed by subsequent IPNV infections of IPNV-resistant farmed salmon led to the emergence of new IPNV variants capable of causing disease in once resistant fish (Benkaroun et al. 2021; Hillestad et al. 2021; Godoy et al. 2022).

Resistance to most diseases that are encountered in swine production is a complex polygenic trait, controlled by a large number of genes (Mallard et al. 2011; Georges et al. 2019), which complicates breeding strategies. Multiple loci have been identified to be associated with resistance to porcine reproductive and respiratory syndrome (PRRS) (Mallard et al. 1992; Wagter-Lesperance and Mallard 2007, You et al. 2023), porcine circovirus type 2 (PCV2)-associated diseases (Eaton et al. 2023), *Haemophilus parasuis* (Fu et al. 2018), and swine influenza virus (Mallard et al. 1998b, Li et al. 2011), but with each only explaining a limited proportion of the genetic variance in resistance or host response to infection.

Several previous studies have aimed to describe the genetic control of the innate and adaptive immune response in pigs. Typically, such studies assign immune-associated phenotypes to individual animals by enumerating white blood cells (leukocytes and lymphocytes) or measuring circulating cytokines and acute phase proteins in blood. Often, vaccination or infection is performed to elicit an immune response prior to the immune phenotype measurement. For example, Flori et al. (2011) examined the genetic control of innate and adaptive immune responses of pigs after vaccination against *Mycoplasma hyopneumoniae*. Similarly, utilizing both production and various measurable immunological components, Uemoto et al. (2021) assigned immune response phenotypes to pigs that were selectively bred over five generations for resistance to *M. pneumoniae* following experimental infection with *M. pneumoniae*. Although both these experiments revealed genetic and genomic associations with immune response, they did so by first challenging the immune system.

Currently, 3,231 quantitative trait loci (QTL) associated with immune response have been identified for the pig, as documented in the QTL data base (Hu et al. 2007). Dauben et al. (2021) also reported that 2,776 QTL have been found for immune response parameters that are measurable in blood and 609 QTL for susceptibility to infectious disease.

Although some genetic basis for immune response and disease resistance has been documented, breeding for pathogen-specific host resistance may increase susceptibility to other pathogens by skewing the host’s adaptive immune system. The adaptive immune response of mammals is complex and can be divided into two branches, the antibody-mediated immune response (AMIR) and the cell-mediated immune response (CMIR), each specialized to support the control of different pathogens. The AMIR is critical to control extracellular pathogens, including many bacterial and parasite species, through the production of various antibody isotypes by B-cells and the action and products (cytokines) of T-helper 2 cells. The CMIR is important for the control of intracellular pathogens, viruses, and facultative intracellular bacteria, and is primarily mediated by T-helper 1 cells and their associated cytokines, and by the activity of T-cytotoxic cells. However, although they represent different branches of the adaptive immune response, AMIR and CMIR participate in coordinated efforts to control infectious disease. Because AMIR and CMIR have been reported to have a negative genetic correlation in cattle (Nino-Soto et al. 2008; Thompson Crispi et al. 2012a, 2012b), targeted breeding for disease resistance, which favors either AMIR or CMIR, rather than both, may increase protection to some pathogens while increasing susceptibility to others. Consequently, a breeding strategy that selects for general, inherent balanced AMIR/CMIR capacity, instead of pathogen-specific resistance, would be advantageous.

Technology to measure AMIR and CMIR on healthy animals, subsequently referred to as the High Immune Response technology (HIR) platform (Wagter-Lesperance and Mallard 2007), was initially developed for pigs (Mallard et al. 1992, 1998b; Wilkie and Mallard 1999) and then translated for use in dairy cattle, where it is now used commercially (Larmer and Mallard 2016). In their initial work, Mallard et al. (1998b) selected Yorkshire pigs over eight generations for high and low immune responsiveness using estimated breeding values for AMIR and CMIR. It was found that the high immune response line pigs had significantly greater antibody responses to various antigens (Magnusson et al. 1997). Additionally, after infection with *Mycoplasma hyorhinis*, the high immune response line pigs had significantly less peritonitis and pleuritis and a higher rate of gain, compared to low and control immune response lines (Magnusson et al. 1998; Mallard et al. 1998b). These initial studies were conducted in controlled research environments using experimental infection with specific pathogens and require validation under commercial conditions with natural infection with multiple pathogens.

In dairy cattle, under commercial conditions, high immune responders have been shown to produce milk of higher quality, as measured by immunoglobulin (Ig) concentration and somatic cell count (Fleming 2014). Wagter et al. (2000) classified Holstein cows in commercial herds based on AMIR phenotype and found high AMIR to be associated with a lower incidence of mastitis, improved response to commercial vaccines, and increased milk and colostrum quality. High-immune-response cows have also been shown to display a lower incidence of other common diseases of dairy cattle, including metritis, ketosis, and retained placenta, and are less susceptible to Johne’s disease (DeLapaz 2008; Pinedo et al. 2009; Thompson-Crispi et al. 2012a). Mallard et al. (1998a) and Thompson-Crispi et al. (2012b) found that differences in AMIR and CMIR have a significant genetic component in dairy cattle and, thus, can be used to breed cows that are more resistant to a diverse array of pathogens (Mallard et al. 2015). Genome-wide association studies (GWAS) in cattle identified the major histocompatibility complex (MHC) region to be important for both AMIR and CMIR (Thompson-Crispi et al. 2014). Genetic and genomic analyses of these HIR traits under commercial conditions have not been conducted in pigs.

Although immune response traits have been found to be heritable in livestock, their genetic relationship with disease resilience, which is the ability of an animal to maintain performance upon exposure to a pathogen and has been recommended as a strategy to breed animals that are better able to withstand pathogen challenges in commercial environments (Knap and Doeschl-Wilson 2020), remains largely under researched.

To investigate the genetic basis of disease resilience in pigs, Putz et al. (2018) and Cheng et al. (2020) described the establishment of a natural disease challenge model (NDCM), in which visually healthy late nursery pigs are exposed to multiple pathogens, including various viruses and bacteria. The challenge model includes collecting an array of resilience phenotypes on individual pigs throughout the challenge period, encompassing growth performance, and clinical disease traits (medical treatment rates, mortality rates, and subjective health scores). The NDCM also provided opportunities to investigate biological traits that can be measured on young healthy pigs as genetic indicators to select for disease resilience, including immune response traits. To this end, AMIR and CMIR, as measured by HIR technology (Schmied et al. 2012), were collected from multiple batches of pigs prior to disease challenge. In a preliminary phenotypic analysis of these data, Schmied et al. (2018) found that pigs classified as high immune responders based on the HIR traits had a lower mortality rate than those classified as low or average when exposed to the natural polymicrobial disease challenge.

Prior studies using data collected in the NDCM did not include a genetic analysis of the HIR data. Thus, using data collected using the NDCM described by Putz et al. (2018) and Cheng et al. (2020), the objectives of this study were to 1) estimate the genetic parameters of HIR traits in young and visually healthy pigs; 2) estimate genetic correlations of HIR traits with disease resilience traits in pigs; and 3) identify genomic regions and genetic markers associated with HIR traits.

## Materials and methods

### Ethical statement

This study was carried out in accordance with the Canadian Council on Animal Care guidelines (*https:((*www.ccac.ca*(en(certification(about-certification*). The protocol was approved by the Protection Committee of the Centre de Recherche en Sciences Animales de Deschambault (and the Animal Care and Use Committee at the University of Alberta (AUP00002227). The project was fully overseen by the Centre de développement du porc du Québec (CDPQ) in Québec, Canada, and its herd veterinarian together with project veterinarians.

### Natural challenge protocol and traits recorded

A natural polymicrobial disease challenge protocol for wean-to-finish pigs was established in 2015 by bringing naturally infected animals into a nursery and finish research facility at the CDPQ, targeting various viral and bacterial diseases. Disease pressure was maintained by entering batches of 60 or 75 visually healthy recently weaned Yorkshire × Landrace barrows, each from one healthy multiplier farm from one of seven breeding companies (members of PigGen Canada), every 3 weeks in a continuous flow system (see Putz et al. 2018 for details), over a period of 5.5 years. Upon arrival at CDPQ, all pigs were vaccinated with a PCV2 vaccine (Circoflex, Boehringer Ingelheim, St. Joseph, MO). The natural challenge protocol consisted of three housing phases: 1) a quarantine nursery (qNur) for 19 days on average, beginning at 3 weeks of age; 2) challenge nursery (cNur) for 27 days on average, where pigs were exposed to the polymicrobial challenge; and 3) a finishing phase (Fin) of 100 days on average, which shared the same air space as cNur. Pigs were re-grouped when moved to cNur and Fin.

In total, disease resilience data on 4,095 pigs from 65 batches over nine cycles (seven batches of pigs comprised one cycle, one from each of the seven breeding companies in rotation) were available. Cycles 7 and 8 of the data used here were separated by 34 batches, during which the standard animal and disease management protocols of the NDCM were continued but that were part of other projects. All data and samples were collected by trained research staff at the CDPQ, using established protocols, as described by Putz et al. (2018) and Cheng et al. (2020). Body weights were obtained on all pigs at entry and exit of the cNur and every 3 weeks in the finisher. Average daily gain (ADG) in the challenge nursery (cNurADG) and the finisher (FinADG) was computed following Cheng et al. (2020). Subjective health scores based on clinical signs were assigned by trained personnel in the quarantine nursery (qNurHS), the challenge nursery (cNurHS), and the finisher (FinHS). Health scores were on a 1 to 5 scale: 1 = severe clinical signs with wasting and 5 = in perfect health. Because of the limited numbers of animals receiving low scores, scores ≤4 were assigned a score of 4 for qNurHS, and scores ≤3 were assigned a score of 3 for the cNurHS and FinHS, as described by Cheng et al. (2020). Clinical treatment rates were adjusted by multiplying the number of treatments a pig received in the corresponding phase by the ratio of the average length of the phase and the number of days the pig spent in the phase and included challenge nursery treatment rate (cNurTRT), finisher treatment rate (FinTRT), and treatment rate across the challenge nursery and finisher (AllTRT). Mortality was recorded as 0 = survived and 1 = died in the challenge nursery (cNurMOR), the finisher (FinMOR), and across the challenge nursery and finisher (AllMOR). For treatment and growth rates, data from pigs that died in the finisher were included in the analyses, with imputation and expansion of treatment and growth rates, as described in Cheng et al. (2020). TRT and MOR phenotypes were analyzed separately for the challenge nursery (cNurTRT and cNurMOR) and for the challenge nursery and finisher combined (AllTRT and AllMOR).

Animals from cycles 1 to 7 were genotyped for 658,692 single-nucleotide polymorphisms (SNPs) with the 650 k Affymetrix Axiom Porcine Genotyping Array by Delta Genomics (Edmonton AB, Canada). The 435,172 SNPs that passed quality control for all seven cycles, as described by Cheng et al. (2020), were utilized for analysis. Animals from cycles 8 and 9 were genotyped with a commercial 50k SNP chip and imputed to the high-density SNP chip using FImpute (Sargolzaei et al. 2014). This was done in two steps. In the first step, 39,525 SNPs on the 50k chip that overlapped with the 435,172 SNPs from the high-density chip were used to impute genotypes of animals from cycles 8 and 9 up to the 435,172 SNPs. In the second step, the 16,217 SNPs on the 50k chip that did not overlap with SNPs on the high-density panel were imputed for the animals that were genotyped for the high-density panel, resulting in genotypes for a total of 451,389 SNPs available on all animals.

### Determination of immune response phenotypes

Using an adapted standard HIR protocol (Schmied et al. 2012), all pigs from cycles 5 to 9 (*n* = 2,295) were phenotyped for HIR traits while in the qNur. To summarize, IR to inert test antigens were used to determine the HIR phenotype of each pig, one that elicits AMIR and one that elicits CMIR (US Patent #7,258,858, Wagter-Lesperance and Mallard 2007). Primary immunization to the combined AMIR and CMIR antigens was made 3 days after piglets arrived in the qNur by intramuscular injection. Blood was collected from each piglet pre-immunization and 2-weeks post-immunization to determine serum antibody activity to the AMIR-associated antigens by enzyme-linked immunosorbent assay (ELISA, Schmied et al. 2012). Cell-mediated IR was evaluated 2-weeks post-immunization by measuring the change in double skin-fold thickness, at the medial aspect of the left inner thigh, 48 hours after intradermal injection of the CMIR-associated antigen compared to change in double skin-fold thickness at the medial aspect of the right inner thigh, 48 hours after intradermal injection of phosphate-buffered saline (PBS) control.

### ELISA protocol

Optimal AMIR antigen-coating conditions were determined using polystyrene, flat-bottomed, Immunolon 2HB, 96-well plates (Fisher Scientific Canada) and 0.05 M carbonate-bicarbonate coating buffer (pH 9.6). Plates were washed three times (405 TS automatic plate washer; Biotek Instruments) with 325 μl 0.05% Tween-PBS (PBST; 0.01 M, pH 7.4, per well). Wells were blocked with 3.0% PBST (pH 7.4), 200 μl per well, and incubated for 1 hour at room temperature (RT). Washing was repeated, and sera diluted in 0.05% PBST were added at 100 μl per well in triplicate. Controls included wells without sera and negative (pooled serum from preimmunized piglets with low antibody activity to the AMIR antigen) and positive (pooled serum from post-immunized piglets with high AMIR antibody activity) sera. Plates were incubated for 2 hours at RT and washed. Alkaline phosphatase conjugated rabbit anti-swine IgG (monoclonal, H + L, Sigma-Aldrich), diluted in 0.05% Tween–Tris-buffered saline (pH 7.4), was added at 100 μl/well and incubated for 1 hour at RT. Plates were washed, and 100 μl *p*-nitrophenol phosphate substrate buffer (Sigma-Aldrich) was added to each well and incubated at RT in the dark. Optical density (OD) of test sample wells was measured at 405 nm (EPOCH; Biotek Instruments) when the positive-control OD reached 1.0. Mean values of OD were obtained from triplicates of test serum and from the controls.

### Genetic parameter estimation

Heritabilities of AMIR and CMIR and phenotypic and genetic correlations between AMIR and CMIR and of AMIR and CMIR with ADG and clinical disease traits were estimated using a GBLUP model in the ASReml software (Gilmour et al. 2022). Because initial analyses identified differences between results from cycles 5 to 7 and 8 to 9, which were separated in time by several months during which the natural disease challenge was continued but used for other purposes, data from cycles 5 to 7 and 8 to 9 were analyzed separately, as well as combined. For estimation of genetic correlations with disease resilience traits, disease resilience data from cycles 1 to 4 were also used when analyzing the HIR data from cycles 5 to 7 and the combined data. The separate analysis of HIR data from cycles 8 to 9 only used the resilience data from those cycles.

The general statistical model for single-trait and bivariate analyses to estimate variance components and genetic correlations was described by Cheng et al. (2020). In summary, fixed effects were batch and the covariate of age when the pig entered the qNur. Random effects were pen by batch, corresponding to the different phases, additive genetic effects, litter environmental effects, and residuals. A genomic relationship with relationships between companies set to zero, in order to obtain pooled within-company variance components, as derived by Cheng et al. (2020), was used for the additive genetic effects. For ADG in the challenge nursery and finisher, and for the finisher and combined treatment rates, whether the pig died during that phase (0/1) was also included as a fixed effect. For AMIR and CMIR, technician (*n* = 11) was also fitted as a fixed effect, along with the AMIR measurement level pre-immunization for AMIR and the CMIR measurement from the saline thigh for CMIR.

### GWAS analyses

For the univariate GWAS of the HIR traits, the marker-based Bayes-B model (Habier et al. 2011) described by Cheng et al. (2022a) was used. This model included the same effects as described above for genetic parameter estimation, except the sum of SNP effects was fitted instead of the animal additive genetic effect. For the SNP effects, a prior probability of 0.1% of a SNP having a non-zero effect (π=0.999) was applied. We chose π = 0.999 because it is difficult to estimate π if the number of individuals is much smaller than the number of markers and these cases, π is usually set equal to =1-(#individuals/#markers) (Serão et al. 2014), which was approximately 0.999 in our case.

The GWAS were implemented using the JWAS package (Cheng et al. 2018), using a Monte Carlo Markov chain of length 50,000, with the first 5,000 iterations discarded as burn-in. The posterior probability of inclusion (PPI) of each SNP was calculated as the proportion of iterations of the chain that the SNP was included in the model with a non-zero effect. Samples of the breeding value of each individual at each saved iteration were calculated as the sum of its SNP genotypes multiplied by the sampled SNP effects for that iteration. The saved samples were also used to estimate the posterior distribution for the genetic variance explained by each non-overlapping 1 Mb window of the reference genome (Sus scrofa 11.1), which was computed as the variance of the window breeding values divided by the total genetic variance in that iteration (Wolc et al. 2012). These posterior distributions were used to estimate the probability of a window having a variance greater than zero (window posterior probability of association, WPPA [Fernando et al. 2017; Wolc and Dekkers 2022]). Genomic regions associated with AMIR and CMIR were declared by identifying non-overlapping 1-Mb genomic windows that were estimated to explain more than 1% of the genetic variance. Candidate genes located in each QTL region were identified in BioMart (Smedley et al. 2009), using the ENSEMBL pig gene database (Sscrofa11.1).

## Results

### Genetic parameters of HIR and genetic relationships with clinical disease traits and growth rate under challenge

Estimates of genetic parameters for AMIR and CMIR based on the GBLUP model for the different combinations of cycles are shown in Table 1. Estimates of heritability were moderately high, consistent across cycles, and slightly greater for AMIR than for CMIR (0.25 vs. 0.20). Estimates for the proportion of phenotypic variance due to litter effects were larger for AMIR (0.06 to 0.17) than for CMIR (0 to 0.05) and, for AMIR, the estimate was substantially larger for cycles 5–7 (0.17 ± 0.04) than for cycles 8–9 (0.06 ± 0.05). The reason for the latter is not clear.

**Table 1:** Estimates (SE) of heritabilities, litter effects (as a proportion of phenotypic variance), and genetic correlations of antibody- (AMIR) and cell- (CMIR) mediated immune response traits in young healthy pigs from different cycles.

Parameter	Trait(s)	Cycles 5–7	Cycles 8–9	Cycles 5–9
**Heritability**	AMIR	0.25 (0.07)	0.20 (0.09)	0.25 (0.06)
	CMIR	0.20 (0.06)	0.20 (0.09)	0.20 (0.05)
**Litter effects**	AMIR	0.17 (0.04)	0.06 (0.05)	0.11 (0.03)
	CMIR	0	0.05 (0.04)	0.02 (0.03)
**Genetic correlation**	AMIR-CMIR	−0.03 (0.20)	0.29 (0.24)	0.23 (0.17)
**Genetic correlation between 5–7 and 8–9**	AMIR-AMIR	0.86 (0.35)		
	CMIR-CMIR	0.91 (0.48)		

The genetic correlation between AMIR and CMIR was essentially zero for cycles 5–7 and moderate for cycles 8–9 (0.29) and for the combined data (0.23). However, none of these estimates were significantly different from zero or from each other. Genetic correlations for AMIR and CMIR between cycles (5–7 vs. 8–9) were high (0.86 and 0.91, respectively) and not significantly different from 1, but with large standard errors (0.35 and 0.48).

**Figure 1:**
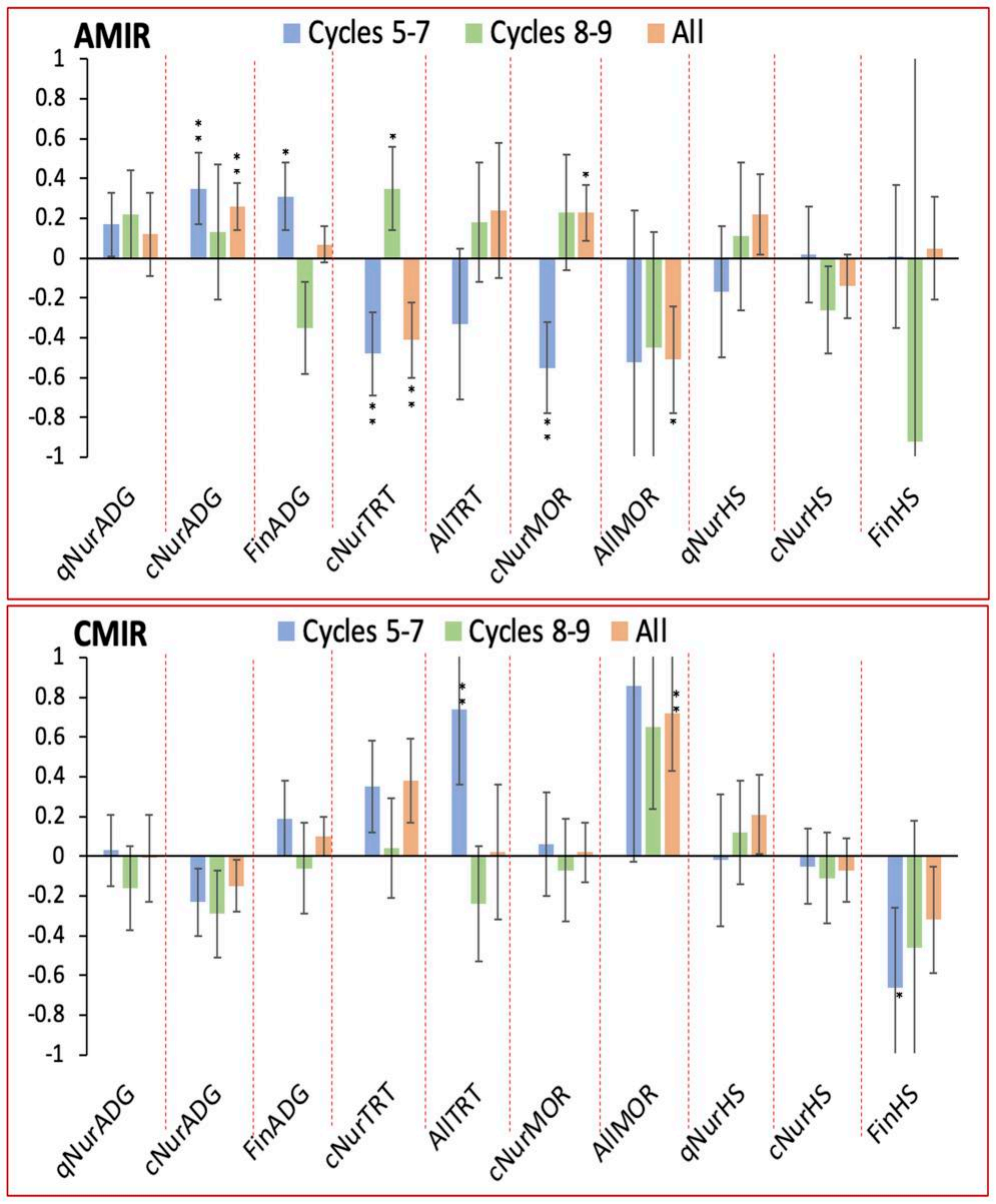
Estimates (and standard error bars) of genetic correlations of antibody- (AMIR) and cell- (CMIR) mediated immune response traits with average daily gain (ADG), veterinary treatment rates (TRT), mortality (MOR), and health scores (HS) in the quarantine nursery (qNur), the disease challenge nursery (cNur), the finisher (Fin), and across the challenge nursery and finisher (All). Separate estimates are shown for analysis of data from cycles 5–7, cycles 8–9, and all cycles. Significance from zero: *P ≤ 0.10. **P ≤ 0.05.

In general, estimates of phenotypic correlations of HIR traits with clinical disease traits and growth rate (not shown) were close to and not significantly different from zero. Estimates of the corresponding genetic correlations were stronger (Figure 1) but also had large standard errors and differed between cycles for a number of traits. AMIR had low but significant (*P* ≤ 0.05) favorable genetic correlation estimates with growth rate in the cNur, driven primarily by its estimate for cycles 5–7. In contrast, CMIR tended to have slight unfavorable genetic correlation estimates with growth rate in the cNur, although none of these estimates were significantly different from zero (*P* > 0.1). Estimates of genetic correlations of HIR traits with ADG in the qNur and finisher were close to zero and differed in sign between the two groups of cycles.

For the clinical disease traits of treatment and mortality rate, AMIR had moderate favorable (ie negative) genetic correlation estimates with TRT and MOR in the cNur for cycles 5–7 (both *P* ≤ 0.05, Figure 1) but these estimates tended to be unfavorable in cycles 8–9 (*P* < 0.1 for cNurTRT and not significant for cNurMOR). Across cycles, this resulted in moderately favorable genetic correlations of AMIR with cNurTRT (-0.41 ± 0.19, *P* ≤ 0.05) and AllMOR (-0.51 ± 0.27, *P* ≤ 0.1) but in small unfavorable genetic correlation estimates with AllTRT (0.24 ± 0.34, not significant) and cNurMOR (0.23 ± 0.14, *P* ≤ 0.1). In contrast, CMIR had moderate unfavorable genetic correlation estimates across cNur and Fin with TRT for cycles 5–7 (0.74 ± 0.38, *P* ≤ 0.05) and with MOR across cycles (0.72 ± 0.28, *P* ≤ 0.05). Estimates for CMIR with TRT and MOR in other phases and cycles were not significantly different from zero but also tended to be unfavorable.

Estimates of genetic correlations of AMIR and CMIR with health scores in the quarantine and cNur were close to zero and not significant (*P* > 0.1) for both AMIR and CMIR (Figure 1). For the health score in the finisher, estimates tended to be negative, in particular for CMIR, and the estimate was significant (*P* < 0.1) for cycles 5–7, implying that higher CMIR was genetically associated with poorer visual health, consistent with some of its genetic correlation estimates with TRT and MOR.

### GWAS for HIR

**Figure 2:**
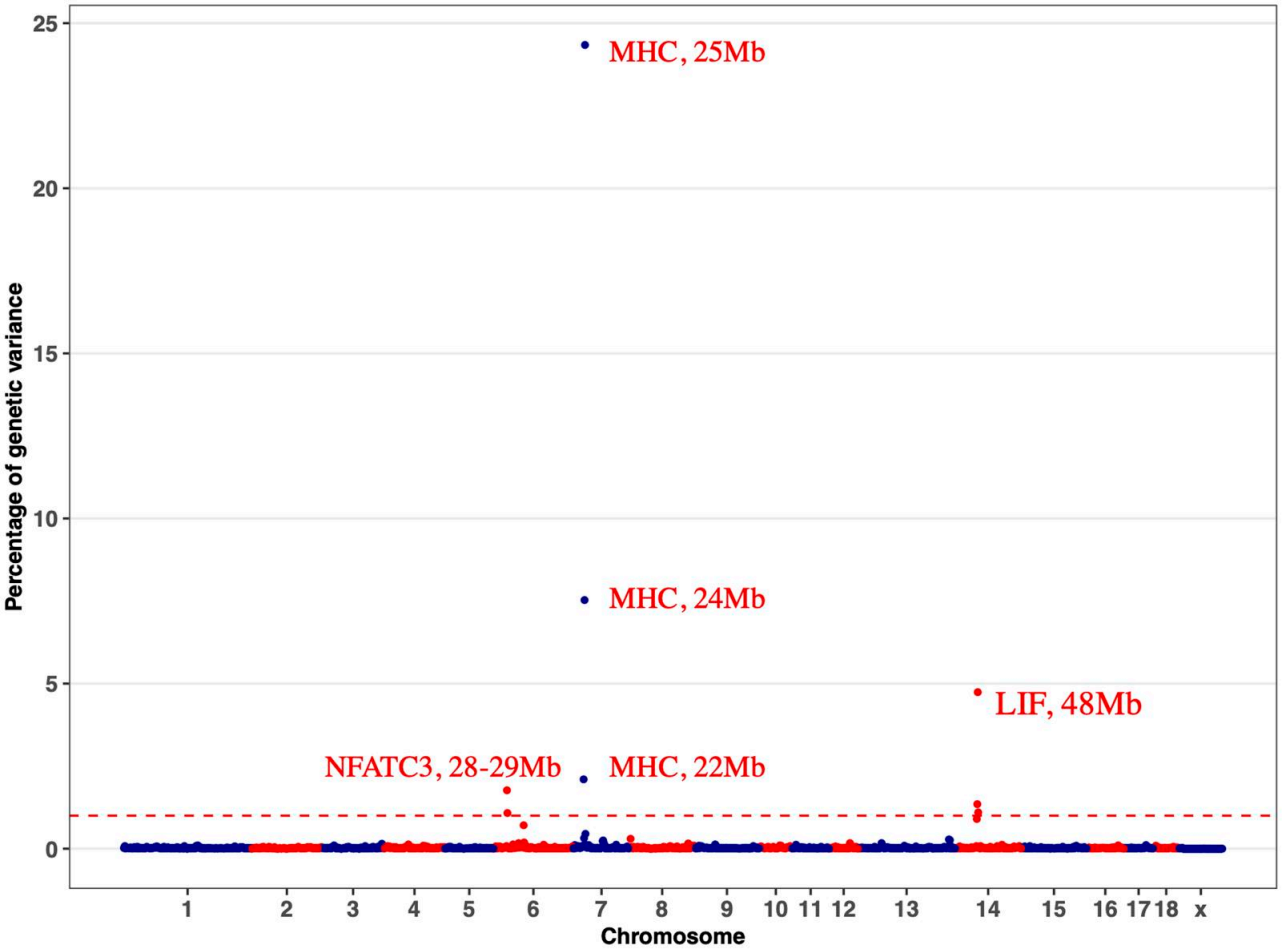
Percentage of genetic variance explained by non-overlapping 1 Mb windows across the genome for antibody-mediated immune response based on the univariate Bayes-B analysis. Windows that explained more than 1% of genetic variance (broken line) were considered significant. MHC, major histocompatibility complex.

**Figure 3:**
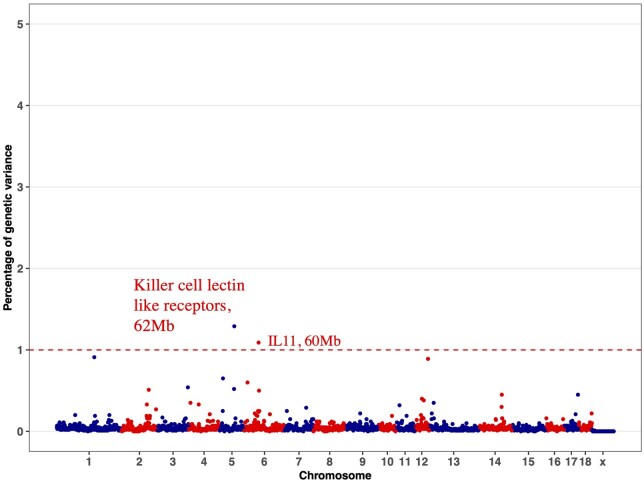
Percentage of genetic variance explained by non-overlapping 1 Mb window across the genome for cell-mediated immune response based on the univariate Bayes-B analysis. Windows that explained more than 1% of genetic variance (broken line) were considered significant.

**Figure 4:**
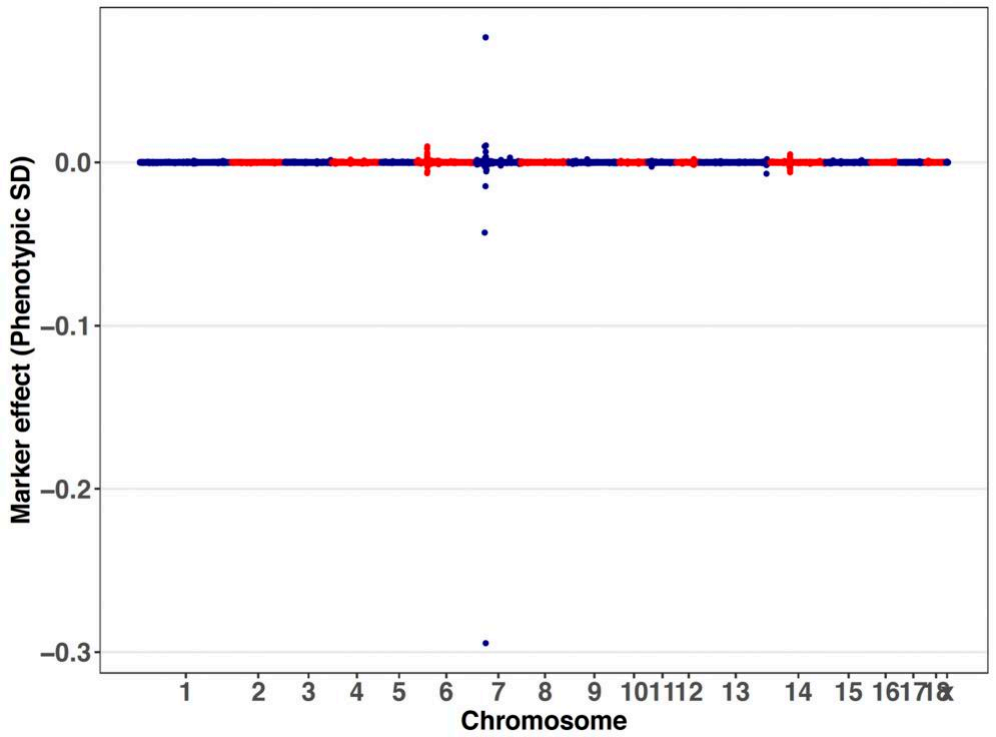
Estimates of marker effects across the genome for antibody-mediated immune response based on the univariate Bayes-B analysis.

Genomic regions identified using univariate GWAS for AMIR and CMIR are shown in Table 2 and Figures 2 and 3. Four genomic regions were detected for AMIR. The two regions on SSC7, at 22 and 24–25 Mb, were located in the MHC region (22–25 Mb) (Hammer et al. 2020) and explained 2.1% and 31.9%, respectively, of the genetic variance, with WPPA of 0.56 and 0.98, respectively. A WPPA of 0.98 provides strong evidence of a QTL for AMIR in the 24–25 Mb region. Within that region, SNP AX-116767878, at 25,170,380 bp, had a very high PPI of 0.98 and the largest estimated marker allele substitution effect (0.3 phenotypic SD, Figure 4). The percentage of genetic variance explained by this SNP was 26%. The genomic regions on SSC6 (28–29 Mb) and SSC14 (47–49 Mb) explained 2.9% and 7.2% of the genetic variance, with WPPA of 0.45 and 0.43, respectively. These two QTL regions harbor the genes *NFATC3* and *LIF*, respectively, which are related to immune response. Two genomic regions were identified for CMIR, at 62 Mb on SSC5 and at 60 Mb on SSC6, which explained 1.3% and 1.1% of the genetic variance, with WPPA of 0.58 and 0.44, respectively. The genomic region on SSC5 harbored several killer cell lectin-like receptor genes, ie *B1, D1, F1*, and *K1*. The genomic region on SSC6 harbors the *IL11* gene, which is related to immune response.

**Table 2:** 1-Mb non-overlapping genomic regions that explained >1% of genetic variance and associated window-based posterior probabilities of association (WPPA) for antibody- (AMIR) and cell- (CMIR) mediated immune responses in young healthy pigs based on a univariate Bayes-B analyses.

SSC	Mb window	% Genetic variance	WPPA	Trait	Candidate genes
**7**	22	2.1	0.56	AMIR	*MHC Class I and III*
**7**	24–25	31.9	0.98	AMIR	*MHC Class III and II*
**6**	28–29	2.9	0.45	AMIR	*NFATC3*
**14**	47–49	7.2	0.43	AMIR	*LIF*
**5**	62	1.3	0.58	CMIR	Killer cell lectin like receptors
**6**	60	1.1	0.44	CMIR	*IL11*

## Discussion

### Measuring immune response capacity

The HIR technology was developed and patented (US Patent #7,258,858) at the University of Guelph (Wagter-Lesperance and Mallard 2007). It was first successfully tested in pigs and globally implemented in 2012 for dairy cattle by Canada’s largest dairy genetics company, the Semex Alliance Inc. under the trade-name, *Immunity+*. The HIR technology measures an animal’s inherent capacity for immune response, capturing the complicated biochemical cascades involved in successful antibody and CMIRs and allows for the identification of animals with both high AMIR and CMIR.

Although AMIR and CMIR represent different branches of the adaptive immune response, they participate in coordinated efforts to control infectious disease. The nature of the pathogen, the dose, the route of infection, and the genetics of the host all influence the balance of AMIR and CMIR. Therefore, challenging pigs with vaccine antigens or infection before or during the establishment of an immune trait phenotype could induce immune response bias, such that the measured immune response parameters are affected by an animal’s antigen-specific immune response, rather than the animal’s overall capacity for a broad-based immune response. As such, focusing on immune response traits after vaccination or infection may reveal only part of the picture if the goal is to breed pigs with broad-based immunity to both viral and bacterial infections. Additionally, immune response to vaccination or infection is complicated by the fact that it is generally impossible to distinguish primary from secondary or later immune responses due to potential previous natural exposure to the common pathogens used in these types of experiments. As a result, a disease challenge experiment may inadvertently compare primary responses in some individuals with memory responses in others. For these reasons, it is better to measure immune responses to antigens that the animal has not previously been exposed to, or when known background responses can be accurately ascertained. Recognizing this, genetic analyses of an animal’s immune response capacity should be conducted using pigs that have not been challenged by a vaccine or a specific infection (Bovo et al. 2019; Dauben et al. 2021), using hematological parameters as phenotypes, including cell counts and blood concentration of cytokines and acute phase proteins. The genetic analyses presented here are unique insofar that they involve measures of immune response without prior vaccination or infection, using the HIR technology.

### Genetic parameters of AMIR and CMIR

The moderately high estimates of heritability of AMIR and CMIR obtained in this experiment of 20% to 25% (Table 1) agree with previous estimates in pigs and in dairy and beef cattle (Mallard et al. 1998b; Thompson-Crispi et al. 2012b; Husseini et al. 2022), and are similar to those for other moderately heritable economically important production traits of swine (Alam et al. 2021). Therefore, genetic improvement of immune response based on HIR is feasible.

In this study, AMIR and CMIR were estimated to have a small positive genetic correlation but it was not significantly different from zero (0.27 ± 0.17). In dairy cattle, Thompson-Crispi et al. (2012b) estimated the genetic correlation between AMIR and CMIR to be negative (-0.13 to -0.45) but also with large SE (0.32 to 0.46). Further research is needed to determine whether the genetic correlation between AMIR and CMIR is fundamentally different between pigs and cattle and/or whether differences are due to the timing and conditions under which the HIR was administered.

For HIR in dairy cattle, GWAS indicated clear differences in genetic control between AMIR and CMIR, with two major QTL identified for AMIR, while QTL associated with CMIR were scattered across the genome (Emam et al. 2019). A similar pattern of genetic control was observed here (Figures 2 and 3).

### Genetic relationships of AMIR and CMIR with clinical disease traits and growth rate under challenge

Both AMIR and CMIR have been shown to have favorable genetic correlations with health in cattle (Thompson-Crispi et al. 2014). In the present study, AMIR was also estimated to be genetically favorably correlated with indicators of health and performance under disease. However, CMIR tended to show unfavorable estimates of genetic correlations with health (Figure 1). These opposing genetic correlations with health between AMIR and CMIR may be explained by the polymicrobial nature of the disease challenge that pigs were exposed to in the present study (Putz et al. 2018; Cheng et al. 2020). As pathogens are diverse, so are the host defense mechanisms that animals use to defend against them. Consequently, components of the immune system may provide protection against certain pathogens, at certain times during an infection, while increasing susceptibility to other pathogens by generating a biased immune response environment. The clinical disease traits that were recorded in the NDCM were primarily focused on bacterial infections. For example, the health trait TRT exclusively described treatments with antibiotics, which are not administered to treat viral disease. Therefore, the unfavorable genetic correlations found between CMIR and TRT and MOR may not be representative of the impact of high CMIR on health. Moreover, although viral diseases were part of the disease challenge, including several strains of PRRS and flu, it should be noted that persistent PRRS infection is a well-established risk factor for secondary infection with respiratory bacteria (Solano et al. 1998). Thus, since the component of the immune system that is captured by CMIR is critical to defense against viral pathogens, it could be hypothesized that pigs with a high CMIR were indeed better able to withstand continual chronic infection with various strains of PRRS, but that this was accompanied by a lack of sufficient AMIR to combat the associated secondary bacterial infections, thereby leading to poor health outcomes (increased treatments with antibiotics) and the observed negative genetic correlations of CMIR with TRT and MOR.

Bishop and Woolliams (2014) stated that “breeding [livestock] for disease resistance is a multi-disciplinary activity.” As such, the observed negative genetic correlation between CMIR and health traits (TRT and MOR) must not be taken strictly at face value. Instead, consideration must be given to the critical immunological impact of CMIR on disease resistance.

To illustrate, in an earlier analysis of phenotypic relationships of AMIR and CMIR with health outcomes in the NDCM by Schmied et al. (2018), a high immune response was found to be associated with lower mortality. Specifically, when pigs were classified based on their AMIR and CMIR phenotypes being low, average, or high, pigs that had a poor or mixed immune response phenotypes died earlier than those who were average or high for both AMIR and CMIR. In addition, piglets classified as poor responders (low-AMIR-low-CMIR, low-AMIR-average-CMIR, and average-AMIR-low-CMIR) died faster and more frequently than piglets of average or mixed response (average-AMIR-average-CMIR, high-AMIR-low-CMIR, and low-AMIR-high-CMIR). Most pigs classified as good responders (average-AMIR-high-CMIR, high-AMIR-average-CMIR) survived longer than those of poor, average and mixed response, reaching market weight more often, and only one of the pigs classified as high-AMIR-high-CMIR died before slaughter (Schmied et al. 2018). Of course, these results were based on phenotypic relationships, whereas we focused on genetic correlations in this study.

Further to the role of CMIR in viral infection, a recent observational study of the Immunity^+^ technology in dairy cattle, revealed that cows with high genomic EBV for both AMIR and CMIR had a lower supportive treatment risk in herds with confirmed outbreaks of highly pathogenic avian influenza than average or low responders (Beard et al. 2026). Moreover, high-immune-response EBV cows had less loss of milk production, an indicator of resilience, during these outbreaks (Beard et al. 2026). Although this observed resistance was attributed to high EBV for both AMIR and CMIR, it is not unreasonable to theorize that enhanced CMIR played a critical role in the response to this virus, given the importance of CMIR in defense against viral pathogens.

Thus, given the emphasis on bacterial infections in the clinical disease outcomes evaluated in the NDCM, and taking the categorical evidence by Schmied et al. (2018) into consideration, as well as previous studies by the Mallard lab and others, showing benefits of both AMIR and CMIR (Magnusson et al. 1997, 1998; Mallard et al. 1998a, 1998b; Thompson-Crispi et al. 2012a, 2012b; Fleming 2014; Mallard et al. 2015; Schmied et al. 2018, Beard et al. 2026), the estimated unfavorable genetic correlations of CMIR with TRT and MOR may not imply that selection for higher CMIR in pigs is deleterious. Indeed, it further emphasizes the importance of AMIR and CMIR being in balance to achieve broad-based disease resistance in complex polymicrobial infection systems, such as those found in commercial swine herds. Furthermore, complex biological and technical factors are at play which may impede estimates of the genetic impact of CMIR on health and production traits, particularly in comparison to AMIR (Emam et al. 2019), including epigenetic regulation of immune response (Mallard et al. 2011).

The different roles of AMIR and CMIR for different pathogens may also explain the different genetic correlations with health and performance traits under disease that were estimated for these two immune response traits between the different cycles (Figure 1). Specifically, AMIR had fairly consistent favorable genetic correlation estimates with growth and health for cycles 5–7 but less so, and even tending to unfavorable genetic correlations, for cycles 8–9 (Figure 1), while CMIR had similar trends but in the opposite direction. The high positive estimates of genetic correlations between cycles for both AMIR and CMIR (Table 1) showed that these differences in genetic correlations of HIR with resilience traits between cycles 5–7 and 8–9 were likely not due to genetic differences in AMIR or CMIR between these two sets of cycles. Instead, the genetic correlation for cNurADG between cycles 5–7 and 8–9 was estimated to be only 0.44 (±0.38), suggesting that the differences in genetic correlations of HIR with resilience traits between cycles were the result of differences associated with the nature of the disease challenge in cycles 5–7 versus 8–9, noting that an additional 34 batches of pigs went through the NDCM between the end of cycle 7 and the start of cycle 8. Putz et al. (2018) listed the pathogens that were identified using passive monitoring during the first 5 cycles of the NDCM. The number of pathogens identified increased over time but this was likely the result of passive monitoring rather than differences in the pathogens that were actually present. In addition, new PRRSV strains were occasionally introduced when infection pressure tended to decrease to levels that prevented expression of differences in disease resilience. However, most differences in infection pressure between batches were likely the result of the dynamic nature of infectious disease related to seasonal differences. The dynamic nature of the disease challenge was investigated and confirmed by Cheng et al. (2022b) for cycles 1–7, who also showed the presence of substantial genotype by environment interactions for disease resilience traits between batches and over time. Such genotype by environment interactions are also expected to be present across the industry. Without the ability to predict the nature of the disease challenge that pigs will encounter in the field, this suggests that the aim of breeding programs should be to breed for pigs that are resilient across the wide array of disease challenge levels and characteristics. Our estimates of genetic parameters across batches in the dynamic natural disease challenge that the NDCM represented provide the parameters that are needed to establish such genetic improvement programs.

As discussed, the immune system is a refined, multi-faceted complex that must maintain the capacity to respond to multiple variable pathogens. Therefore, selecting on individual components of the adaptive immune system or in opposite directions (ie AMIR vs. CMIR) can endanger this critical balance. Although on the aggregate, results of this study suggest that selecting for a combination of higher AMIR and lower CMIR may improve resilience, account must be taken of the known biological importance of balanced AMIR and CMIR when the breeding goal is broad-based disease resistance. With proper weights on these two traits in a balanced selection program for productivity, efficiency, and disease resilience, changes in AMIR and CMIR are not expected to be so dramatic that there is a danger that the different components of the immune system are pulled out of balance, but this does need to be monitored.

### Genome-wide association study

Our GWAS studies found that the 25 Mb region of SSC7 was strongly associated with AMIR (Figure 2), and the majority of the effect of this QTL was captured by one SNP. This is the MHC II region and contains the MHC class II series of genes of the adaptive immune system that are related to antigen presentation and initiation of the adaptive immune response. Research conducted by Thompson-Crispi et al. (2014) provided the first GWAS for general adaptive immune response in dairy cattle, revealing variation in SNP profiles between cows classified as high and low immune responders by HIR technology. This work was the foundation of the successful development of a genomic test for immune response in dairy cattle (Wagter-Lesperance and Mallard 2007) and revealed a cluster of significant SNPs on chromosome 23 in cattle, the location of MHC II in cattle, and associated with AMIR (Thompson-Crispi et al. 2014). In our analysis, the SNP in the MHC Class II region explained 26% of the genetic variance for AMIR. Thus, a similar genomic test for immune response in swine could be developed based on our findings. MHC class I and III regions were also found associated with AMIR in this study, at 22 and 24 Mb, respectively (Figure 2). These regions harbor the MHC class I series of genes of the adaptive immune system, as well as key immunity-related genes that are important for immune defense mechanisms and inflammation (Hammer et al. 2020). Several previous studies have identified similar strong associations of the MHC with antibody response in pigs based on sample-to-positive (S/P) ratio for anti-PRRSV IgG in the blood of sows following a natural PRRS outbreak (Serão et al. 2014; Hickmann et al. 2021) or PRRS vaccination (Sanglard et al. 2020, 2021). Our previous GWAS on disease resilience traits from this study also identified strong associations of the MHC II region with growth rate and health in these data (cycles 1–7) (Cheng et al. 2022a). The bivariate GWAS analyses between AMIR and growth and health traits (results not shown) also suggested that there is a pleiotropic QTL in the MHC II region that is associated with AMIR, growth rate, treatment and mortality rate, and health score.

The QTL that was identified on SSC6 for AMIR (Figure 2) harbors the gene *NFATC3*, which is a member of the nuclear factor of activated T-cells (NFAT) family and has been found to be important in the regulation of genes involved in T-cell activation (Urso et al. 2011). The QTL identified on SSC14 for AMIR harbors the gene *LIF*, which has a pivotal role in T-cell immunity (Metcalfe 2011). These genes, coupled with the MHC genes, are probably working together during the AMIR response to antigens.

The QTL detected for CMIR (Figure 3) harbors several killer cell lectin-like receptors and the *IL11* gene. Killer cells are involved in immune response (Yokoyama and Plougastel 2003), while *IL11* is a member of the glycoprotein-130 (GP-130) cytokines that utilizes the GP-130 signaling pathway shared by other cytokines of the same family. It is an anti-inflammatory cytokine but, depending on the context, can also act as a proinflammatory cytokine, suggesting a complex role in immune response (Xu et al. 2016). Overall, the QTL identified for CMIR had smaller estimated effects than those identified for AMIR. Previous studies have also noted that CMIR has multiple QTL with small effects (Thompson-Crispi et al. 2014). This is consistent with the nature of CMIR, which involves many cell types that infiltrate the test site, as compared to AMIR, which measures antibodies that are produced only by B-lymphocytes (Emam et al. 2019).

## Conclusions

Both AMIR and CMIR were moderately heritable and showed evidence of genetic correlations with performance and health traits under a severe and dynamic natural polymicrobial disease challenge. This suggests that they are strong candidates as indicator traits that can be measured on young healthy pigs to generate correlated responses in resilience across multiple pathogens. AMIR was estimated to have favorable genetic correlations with growth and health under disease, but CMIR showed unfavorable genetic correlations with health, which may relate to the nature of evaluated clinical disease outcomes and will need to be validated in commercial herds. GWAS identified an important SNP for AMIR in the MHC II region, AX-116767878, which was found to explain 26% of the genetic variation for AMIR. Selection based on this SNP can, therefore, be incorporated into pig breeding programs through marker-assisted selection, with the goal of improving disease resilience.

## Data avaliability

The data analyzed in this study were collected on animals that were provided by and are part of the commercial breeding programs of the 7 investing member businesses of PigGen Canada (https://piggencanada.org/, last accessed 12/30/2021). As such, the data and samples generated on these animals are confidential and protected as intellectual property or as trade secrets. As a result, the data analyzed in this study are not publicly available but are stored in a secure data base at the University of Alberta. Data can, however, be made available on reasonable request.

*Conflict of interest statement*. Members of PigGen Canada, ie the seven breeding companies, provided the pigs whose data were used in this study, and geneticists of the companies were involved in the design of the natural disease challenge project. However, because of the design of the study, this did not lead to actual conflicts of interest. First, members of PigGen Canada were not involved in the management of pigs under the challenge, nor in the data analysis, or interpretation of results. In addition, because of the confounding between batch and company (pigs from a given batch originated from one company) and the dynamic nature of the disease challenge, by design, the study did not enable comparisons between companies to be made, which could lead to conflicts of interest. Instead, the entire focus was on analysis of differences between pigs within a batch, ie on genetic differences among pigs within a company. None of the other co-authors had real or perceived conflicts of interest.

## Abbreviations

ADFI: average daily feed intake
ADG: average daily gain
All: across the challenge nursery and finisher
AMIR: antibody-mediated immune response
CMIR: cell-mediated immune response
cNur: challenge nursery
EBV: estimated breeding value
ELISA: enzyme-linked immunosorbent assay
Fin: finisher
GWAS: genome-wide association study
HIR: high immune response
HS: health score
Ig: immunoglobulin
MHC: major histocompatibility complex
MOR: mortality
PBS: phosphate-buffered saline
PCV: porcine circovirus
PPI: posterior probability of inclusion
qNur: quarantine nursery
QTL: quantitative trait locus
SNP: single-nucleotide polymorphism
S/P ratio: sample-to-positive ratio
TRT: treatment rate
WPPA: window posterior probability of association
